# Incidental finding at methylation-specific multiplex ligation-dependent probe amplification (MS-MLPA): how to proceed?

**DOI:** 10.3389/fgene.2023.1274056

**Published:** 2023-10-03

**Authors:** Africa Manero-Azua, Arrate Pereda, Isabel Llano-Rivas, Intza Garin, Guiomar Perez de Nanclares

**Affiliations:** ^1^ Rare Diseases Research Group, Molecular (Epi) Genetics Laboratory, Bioaraba Health Research Institute, Araba University Hospital-Txagorritxu, Vitoria-Gasteiz, Araba, Spain; ^2^ Service of Genetics, Hospital Universitario Cruces, Barakaldo, Bizkaia, Spain

**Keywords:** incidental findings, MS-MLPA, reference probe, Silver-Russell syndrome, 22q11.2 deletion syndrome

## Abstract

**Introduction:** Since the advent of new generation sequencing, professionals are aware of the possibility of obtaining findings unrelated to the pathology under study. However, this possibility is usually forgotten in the case of studies aimed at a single gene or region. We report a case of a 16-month-old girl with clinical suspicion of Silver-Russell syndrome (SRS).

**Methods:** Following the international SRS consensus, methylation alterations and copy number variations (CNVs) at 11p15 region and maternal uniparental disomy of chromosome 7 were analysed and discarded by MS-MLPA.

**Results:** Unexpectedly, the 11p15 region MS-MLPA showed a decrease in the signal of a copy number reference probe. Deletions affecting a single probe are inconclusive. So, we faced the ethical dilemma of whether it was appropriate to confirm this alteration with independent techniques and to offer a diagnostic possibility that was in no way related to clinical suspicion. Fortunately, in this particular case, the informed consent had not been specific to a particular pathology but to any disorder associated with growth failure. Performed alternative studies allowed the final diagnosis of 22q deletion syndrome.

**Conclusion:** We demonstrate the importance of informing patients about the possibility of obtaining incidental findings in genetic techniques (not only in next generation sequencing) during pre-test genetic counselling consultations. In addition, we highlight the relevance of including in the informed consent the option of knowing these unexpected incidental findings as in some cases, this will help to elucidate the definitive diagnosis and provide the correct follow-up and treatment.

## 1 Introduction

Not so long ago targeted sequence analysis of single genes was performed to identify the genetic causative variant for a specific disease. With the implementation of whole exome sequencing as a first-tier test (and even genome sequencing in some countries), analysis is extended to all protein-coding genes, and consequently, the probability of detecting unexpected and/or unsolicited findings has increased (Crawford et al., 2013; Shkedi-Rafid et al., 2014). These non-required results have been divided into secondary findings and incidental findings. Even both of them are (likely) pathogenic variants not related to the initial clinical question, secondary findings refers to those variants located at genes that are *actively looked for* by the clinical laboratory (Lazier et al., 2022) and whose search is based on the list proposed by the American College of Medical Genetics (Miller et al., 2022). On the other hand, incidental findings include (likely) pathogenic variants not related to the primary clinical indication that are identified *by chance* during the genetic analysis (Lazier et al., 2022).

While the first recommendations for secondary findings were proposed in 2013 (Green et al., 2013) and have been updated and adapted in subsequent proposals (Kalia et al., 2017; Miller et al., 2021; Miller et al., 2022), the same has not been true for incidental findings since 2011 (Berg et al., 2011). In fact, there is lack of consensus about whether or not genetic incidental findings should be automatically disclosed to patients (Hegde et al., 2015). This aspect is even more difficult to address when the genetic study is to be carried out in children (Wilfond and Carpenter, 2008; Anderson et al., 2015; Saelaert et al., 2018; Sergi et al., 2023). Even more, if trio exome sequencing is performed, these unsolicited variants, when found in the patient, could be included in the report and add information regarding inheritance for variants, and thus has the potential to diagnose a parent at the same time as a child. Even if family members do not undergo sequencing, the identification of an incidental finding in a child can have implications for the entire family, because cascade testing may be recommended for unaffected family members. This implication of the results of children’s genetic studies on their parents could have an effect on the parents’ decision on whether or not to carry out the genetic study (Sergi et al., 2023).

Despite all the progress that has been made in relation to ethical aspects and informed consents in new genomic technologies, these features do not seem to have been covered in other, “conventional,” technologies. To the best of our knowledge there is nothing similar planned in studies targeting not only the gene/chromosomal region of interest but also the use of reference elements such as at fluorescence *in situ* hybridization (FISH), Multiplex Ligation-dependent Probe Amplification (MLPA) or methylation specific (MS)-MLPA. So how should we act in the face of unexpected/incidental findings?

In this paper we describe how we deal with incidental findings in a girl after an MS-MLPA study.

## 2 Patient and methods

### 2.1 Case report

**FIGURE 1:**
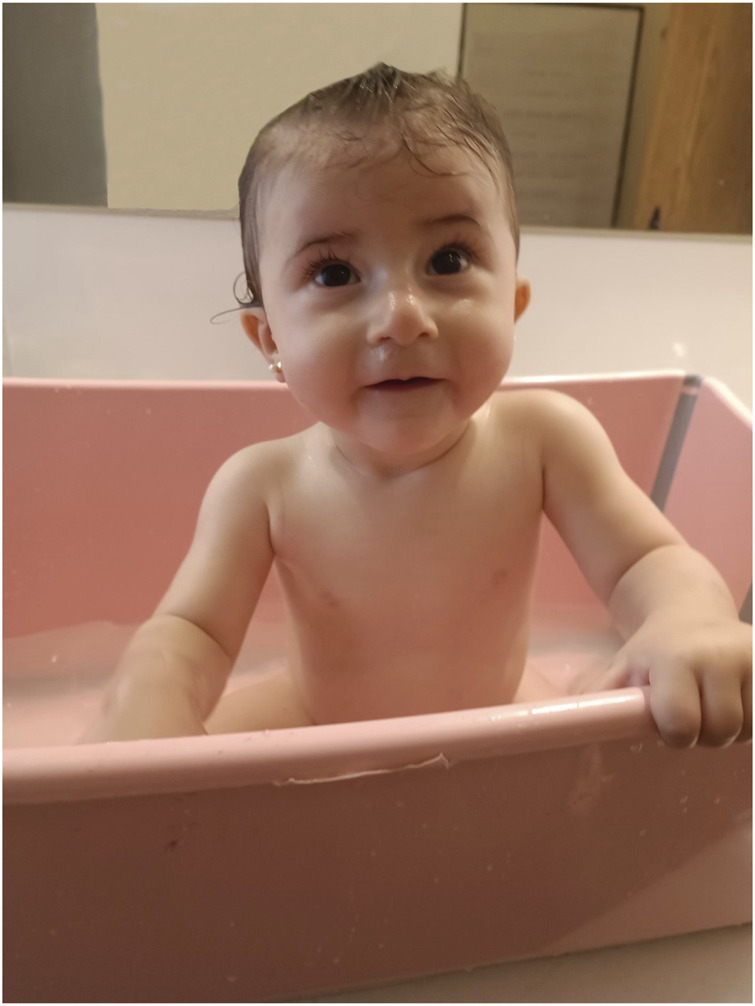
Photography of the front view face of the patient facial signs. Initial clinical suspicion was based on the presence of a high forehead and a triangular-shaped face.

A 16-months-old girl was referred by the Digestive Service for genetic testing of Silver-Russell syndrome (SRS) based on short stature and facial signs resembling this syndrome (Figure 1). Delivery was induced at 37 + 4 weeks of gestation due to weight and height stagnation. Her height was 72.5 cm (<p3) and her weight 7.68 kg (<p3). Parents reported feeding difficulties and hypotonia.

Clinical genetics re-evaluation at 20 months of age confirmed growth failure (height 76 cm, <p3, -2.54 SD) and weight below 3^rd^ centile (9.02 kg, <p3) with microcephaly (head circumference 43.8 cm, <p3 −3.66 SD). Exhaustive phenotype examination revealed short, downslanting palpebral fissures; prominent nasal bridge, wide nasal root and small nostrils; small mouth; nasal speech and food leakage through the nose compatible with velopharyngeal incompetence and inexpressive facies. Echocardiographic evaluation discarded structural alterations.

### 2.2 SRS clinical testing by MS-MLPA

After genomic DNA extraction using QIAamp Blood Mini (Qiagen, Düren, Germany), and following the SRS consensus statement (Wakeling et al., 2017), methylation alterations and copy number variations at 11p15 and chromosome 7 were analysed. The 11p15 chromosomal methylation pattern was measured by the SALSA MLPA Probemix ME030-C3 BWS/RSS (lot: C3-0121, MRC-Holland, Amsterdam, Netherlands) which interrogates the IC1 (*H19/IGF2:*IG-DMR) and IC2 (*KCNQ1OT1:*TSS-DMR) domains. Afterwards, maternal uniparental disomy of chromosome 7 (upd(7)mat) was assessed by using the SALSA MLPA Probemix ME032 UPD7-UPD14 (lot: B1-0921, MRC Holland). Both MS-MLPA tests were performed following the manufacturer´s instructions.

### 2.3 Analyses for confirming the 22q11 deletion syndrome

The study of a possible deletion at the 22q11 region was performed by MLPA with the P250 DiGeorge kit (lot: B2-0519, MRC Holland).

In order to confirm and establish the extension of the detected CNV, a comparative genomic hybridization oligonucleotide microarray (aCGH), containing around 60,000 probes distributed throughout the genome (60 K from Agilent qChip®Post CM kit; qGenomics; Agilent Technologies, Santa Clara, CA) was used. Test sample was hybridized against a sex-matched reference (human reference DNA, Agilent Technologies). Data normalization was carried out with standard settings of the Feature Extraction software and afterwards were analysed using Cytogenomics 4.0.3.12 and qGenviewer software (analysis parameters: algorithm ADM2 ≥ 6.0; abs (log2ratio) ≥ 0.25; probes ≥3).

## 3 Results

**FIGURE 2:**
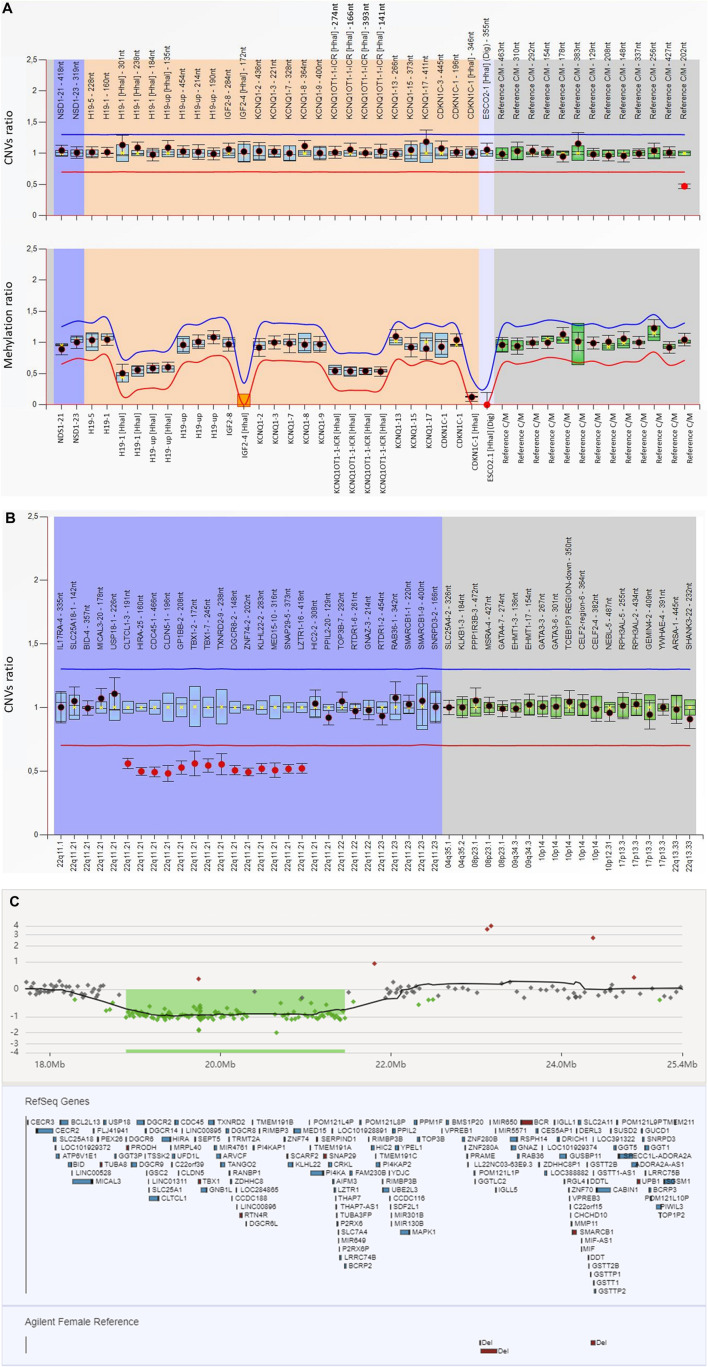
Results of the molecular studies **(A)** The MS-MLPA analysis for 11p15 (ME030-C3 BWS/RSS; lot: C3-0121) with Coffalyser software revealed a decreased signal of a reference probe located at 22q11 (Reference C/M 22–019,079,440-202 nt). For the generation of these results, the software does an intra-normalization, converting absolute fluorescence signal intensities into relative values by normalizing probe signals against the signals of the reference probes in one sample. This is done for each sample. During inter-normalization, it compares each sample to the reference samples. In the present figure, the name of the analysed probes are given on the *X*-axis. The orange background encompasses the probes located at 11p15; the dark blue the *NSD1* gene, involved in Sotos syndrome, a differential diagnosis for BWS/SRS; and the gray one covers the reference probes which are located on different chromosomes and are used for dosage normalization. The 95% confidence interval over the reference samples for each probe is represented by the blue square. The border lines (red for lower border; blue for upper border) are placed −/+ 0.3 from the average probe value of a probe over the reference samples. When a probe is within the borders, it is represented by a black dot; when it is out (either over the blue or below the red lines) it is represented by a red dot. **(B)** MLPA analysis targeting 22q11 (P250 DiGeorge (lot: B2-0519) performed after revision of the informed consent confirmed the presence of a heterozygous deletion at region 22q11. The blue background encompassed the analyzed probes through the 22q11 region whereas the grey background covers the reference probes. The analysed probes at 22q11 region and intra normalized ratio for CNVs are given on the *X*-axis and *Y*-axis, respectively. Probe ratios are indicated by the dots. Black dots indicate the probe ratio is within the 95% confidence interval (CI) of the reference sample population and the red dots indicates the ratio is out of the 95% CI and over the arbitrary borders, lower border: red line (0.7) upper border: blue line (1.3), by default. The boxes represent the 95% CI in reference sample population (by default), the blue boxes compared to test probes and the green ones compared to reference probes. **(C)** Array CGH analysis of the patient showed a deletion of 2.5 Mb at 22q11.21: arr [GRCh37] 22p11.21 (18894864–21461811)x1, encompassing approximately 40 genes.

No (epi)genetic alterations were found for chromosome 7, chromosome 14q32, nor chromosome 11q15, which are the main underlying known molecular mechanisms of SRS. However, in the analysis of the assay done by BWS/RSS MS-MLPA we observed a 50% decreased signal of a copy number reference probe located at 22q11 (Reference C/M 22-019,079,440-202 nt). The MS-MLPA test was repeated and results were confirmed, suggesting that the patient may harbor a heterozygous deletion in this region (Figure 2A).

Even if the clinical suspicion was SRS, the family had signed an informed consent for any disorder related with short stature. Subsequently, the MLPA targeting 22q11 was performed and the presence of a heterozygous deletion encompassing, at least, from exon 3 of *CLTCL1* to exon 16 of *LZTR1* was confirmed (Figure 2B). Parental analyses confirmed the *de novo* origin (data not shown).

The aCGH analysis confirmed the existence of a pathogenic interstitial deletion of approximately 2.8 Mb (arr [GRCh37] 22q11.21 (18894864–21461811)x1) in the 22q11.21 chromosomal band (Figure 2C).

## 4 Discussion

Silver-Russel Syndrome (SRS, OMIM#180860) is a rare genetic imprinting disorder associated with prenatal and postnatal growth retardation. Due to the heterogenous clinical manifestations within SRS patients, the clinical diagnosis is currently based on the Netchine-Harbison clinical scoring system (NH-CSS), including the following six main clinical criteria: 1) born small for gestational age, 2) postnatal growth restriction, 3) relative macrocephaly at birth, 4) prominent forehead at 1–3 years; 5) body asymmetry and 6) feeding difficulties and/or low body mass during early childhood (Azzi et al., 2015). The recent international consensus statement for SRS suggests the genetic testing for the known molecular alteration in patients scoring 4 or more of 6 factors (Wakeling et al., 2017).

The most common genetic underlying mechanisms in SRS patients are hypomethylation of IC1 (*H19/IGF2:*IG-DMR), on chromosome 11p15 (seen in 30%–60% of patients) and maternal uniparental disomy for chromosome 7 (upd (7) mat; seen in ∼5–10% of patients) (Wakeling et al., 2017). In 1%–2%, alterations at 14q32 can be identified, corresponding to molecular findings associated with another imprinting disorder, Temple syndrome (MIM# 616222) (reviewed in Eggermann et al., 2015). Although different techniques are available for the analysis of the methylation status, the most regularly used molecular assay is the MS-MLPA (Mackay et al., 2022).

The MS-MLPA assay (Nygren et al., 2005) detects both genetic (CNVs) and epigenetic (DNA methylation) disturbances. It includes three type of probes: 1) those designed to detect CNVs at the region(s) of interest; 2) those designed to determine the methylation status of those regions; and 3) reference probes used for the normalization at the dosage analysis. According to the manufacturer, a reference probe is the one that detects a sequence that is expected to have a normal copy number in (almost) all samples (MRC Holland, 2022).

In the present case, even if the patient did not completely fulfil the NH-CSS criteria (Table 1), her youth and the presence of additional clinical features (Wakeling et al., 2017) prompted us to perform the genetic studies associated with this pathology. Even if no molecular alteration was detected at chromosome 7, 14q32 nor 11p15, we identified a decrease of intensity on a copy number reference probe located at 22q11. According to the manufacturer, copy number changes detected by a single probe always require confirmation as a lowered probe signal can be due to the presence of a mutation/polymorphism.

**TABLE 1:** Description of the clinical features associated with SRS according to the international consensus (adapted from Wakeling et al., 2017, licensed CC-BY-4.0), their presence or absence in the present case and in another previously reported patient also misdiagnosed with SRS and carrying the 22q11 deletion (Spengler et al., 2012).

	Present patient	SRS1251/06
**NH-CSS main clinical diagnosis**		
1. Small for gestational age (birth weight and/or birth length)	+	ND
2. Postnatal growth failure	+	+
3. Relative macrocephaly at birth	-	+
4. Prominent forehead	?	ND
5. Body asymmetry	-	ND
6. Feeding difficulties and/or low BMI	+	ND
**Additional clinical features for SRS**		
Triangular face	+	+
Fifth finger clinodactyly	-	+
Shoulder dimples	-	-
Micrognathia	-	-
Low muscle mass	+ (hypotonia)	-
Excessive sweating	ND	ND
Low-set and/or posteriorly rotated ears	-	-
Down-turned mouth	-	+
High pitched or squeaky voice	-	-
Prominent heels	-	-
Delayed closure of fontanelle	ND	-
Male genital abnormalities	ND	-
Speech delay	-	+
Irregular or crowded teeth	-	+
Motor delay	-	ND
Syndactyly of toes	-	-
Hypoglycaemia	-	-
Scoliosis and/or kyphosis	ND	-

SGA, Small for gestational age; BMI, Body mass index; ND, no data.

But, could we go ahead and confirm if there is a polymorphism or a real deletion at 22q11 in the present patient? As the manufacturer indicates, copy number changes detected by reference probes or flanking probes are unlikely to be related to the condition tested for. The chromosome 22q11.2 deletion syndrome (OMIM#611867) and SRS share some clinical manifestations such as the short stature, feeding problems and speech delay. In fact, this is not the first time of misdiagnosis among these two entities (Table 1) (Spengler et al., 2012). Nevertheless, even a specific MLPA kit exists to confirm (or discard) the presence of deletions at 22q11.2, could we analyse this region without informing the family? Or, should we inform the family of the possibility of such a syndrome, not clinically suspected, which technically we could not confirm given that we had only identified a single altered probe? Or, should we inform the family regarding this incidental finding if no consent on this point was recorded as nobody thought on the possibility of incidental finding as a result of an MS-MLPA? Or, should we confirm the possible deletion by alternative techniques and, depending on the results, inform the family?

Fortunately, in our case, the signed informed consent was not specific for SRS. In fact, the family accepted the study of any disease/syndrome associated with short stature. This study was the one that the family was asked about in the pre-test counselling consultation since, as mentioned above, the child did not fully meet the criteria for clinical suspicion of SRS, despite her young age. Based on that, MLPA test targeting 22q11 was run and confirmed the presence of a heterozygous deletion whose boundaries were defined by a posterior aCGH. In particular, the patient carries the most prevalent microdeletion in humans, the proximal A-D deletion (LCR22A-D) (Burnside, 2015). According to the clinical and molecular findings, the diagnosis of chromosome 22q11.2 deletion syndrome (most probably the velocardiofacial syndrome) was established. The family was informed. The communication of the final diagnosis to the parents was emotionally impactful for them, not only because they had another child, but also because of the severity of the common clinical manifestations of the 22q11.2 deletion syndromes (Putotto et al., 2022) and the profile of other patients related with the disease. Happily, none of the parents was carrier of deletion, so the disease was ruled out in the patient’s brother. Moreover, the girl does not suffer any cardiac anomaly and does not seem to present cognitive/behavioral issues (even if under follow-up by neuropaediatricians for assessment of progress) and henceforth, clinical management and treatment are guided to her final diagnosis.

In view of the mixed reactions to incidental findings and the often emotional and psychological impact on families (Carrasco et al., 2022; Cheung et al., 2022), we emphasize the importance of addressing these possible unexpected genetic results in pre-test genetic counselling, even when performing classical genetic techniques. In addition, informed consent should include the option of choosing whether they wanted to receive them.

## 5 Conclusion

With this work our intention has been to show that incidental findings can also be found when using genetic (not only genomic) techniques and that this fact should be taken into account in our pre-test genetic counselling consultations.

## Data Availability

The raw data supporting the conclusion of this article will be made available by the authors, without undue reservation.
